# Advances and applications of fluids biomarkers in diagnosis and therapeutic targets of Alzheimer's disease

**DOI:** 10.1111/cns.14238

**Published:** 2023-05-05

**Authors:** Yanan Xu, Hailun Jiang, Bin Zhu, Mingnan Cao, Tao Feng, Zhongshi Sun, Guanhua Du, Zhigang Zhao

**Affiliations:** ^1^ Department of Pharmacy, Beijing Tiantan Hospital Capital Medical University Beijing China; ^2^ School of Pharmacy Capital Medical University Beijing China; ^3^ Center for Movement Disorders, Department of Neurology, Beijing Tiantan Hospital Capital Medical University Beijing China; ^4^ Department of Pharmacy The Sixth Medical Center of PLA General Hospital Beijing China; ^5^ The State Key Laboratory of Bioactive Substance and Function of Natural Medicines Beijing China; ^6^ Key Laboratory of Drug Target Research and Drug Screen, Institute of Materia Medica Chinese Academy of Medical Science and Peking Union Medical College Beijing China

**Keywords:** Alzheimer's disease, biomarkers, blood, cerebral spinal fluid, saliva, treatment

## Abstract

**Aims:**

Alzheimer's disease (AD) is a neurodegenerative disease with challenging early diagnosis and effective treatments due to its complex pathogenesis. AD patients are often diagnosed after the appearance of the typical symptoms, thereby delaying the best opportunity for effective measures. Biomarkers could be the key to resolving the challenge. This review aims to provide an overview of application and potential value of AD biomarkers in fluids, including cerebrospinal fluid, blood, and saliva, in diagnosis and treatment.

**Methods:**

A comprehensive search of the relevant literature was conducted to summarize potential biomarkers for AD in fluids. The paper further explored the biomarkers' utility in disease diagnosis and drug target development.

**Results:**

Research on biomarkers mainly focused on amyloid‐β (Aβ) plaques, Tau protein abnormal phosphorylation, axon damage, synaptic dysfunction, inflammation, and related hypotheses associated with AD mechanisms. Aβ_42_, total Tau (t‐Tau), and phosphorylated Tau (p‐Tau), have been endorsed for their diagnostic and predictive capability. However, other biomarkers remain controversial. Drugs targeting Aβ have shown some efficacy and those that target BACE1 and Tau are still undergoing development.

**Conclusion:**

Fluid biomarkers hold considerable potential in the diagnosis and drug development of AD. However, improvements in sensitivity and specificity, and approaches for managing sample impurities, need to be addressed for better diagnosis.

## Graphical abstract

From brain to periphery: changes in proteins, lipids, and RNA molecules characteristic of the neurodegenerative process may suggest early lesions and become targets for intervention.

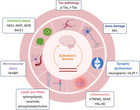

## 1 INTRODUCTION

Alzheimer's disease (AD) is the most common cause of dementia and is characterized by declining cognition and memory. The defects in cholinergic neurons indicate the primary feature representing AD. These include the degeneration of cholinergic neurons in the basal nucleus of Meynert,^1^ the decline of choline acetyltransferase activity,^2^ and the reduction of cholinergic basal cortical projection.^3^ The two typical pathologies of AD involve the formation of senile plaques by amyloid β peptide (Aβ) deposition and neurofibrillary tangles (NFT) by hyperphosphorylation of Tau protein.^4^ This could lead to the subsequent death of neurons. However, the underlying mechanism behind neurodegeneration, neurotoxicity, and cognitive impairment remains unclear.

According to these findings, cholinesterase inhibitors were first developed to treat AD.^5^ Three cholinesterase inhibitors are currently approved for AD: donepezil, galanthamine, and rivastigmine. Additionally, memantine, which can regulate glutamate, dopamine, serotonin, acetylcholine, and other excitatory neurotransmission, was extensively used in AD patients. In June 2021, a novel drug called aducanumab (trade name “Aduhelm”) was approved by the Food and Drug Administration (FDA) in the United States. This was followed by the approval of lecanemab (trade name “Leqembi”) in January 2023. Aducanumab and lecanemab are immunoglobulin gamma 1 monoclonal antibodies selectively binding to Aβ fibrils and soluble oligomers.^6^, ^7^ These drugs provide a new treatment strategy, representing a breakthrough in AD treatment.

The above six drugs are the only approved therapeutic drugs against AD. Other than the controversial aducanumab and just‐approved lecanemab, the therapeutic results of the other drugs also remained unsatisfactory. Since these drugs could not prevent neuronal degeneration, although they improved the clinical symptoms and quality of life of patients during the initial treatment stages. The biggest challenge is the short‐term efficacy of these drugs, which is approximately 1 ~ 3 years, and cannot prevent the disease from progressing.^1^

**FIGURE 1:**
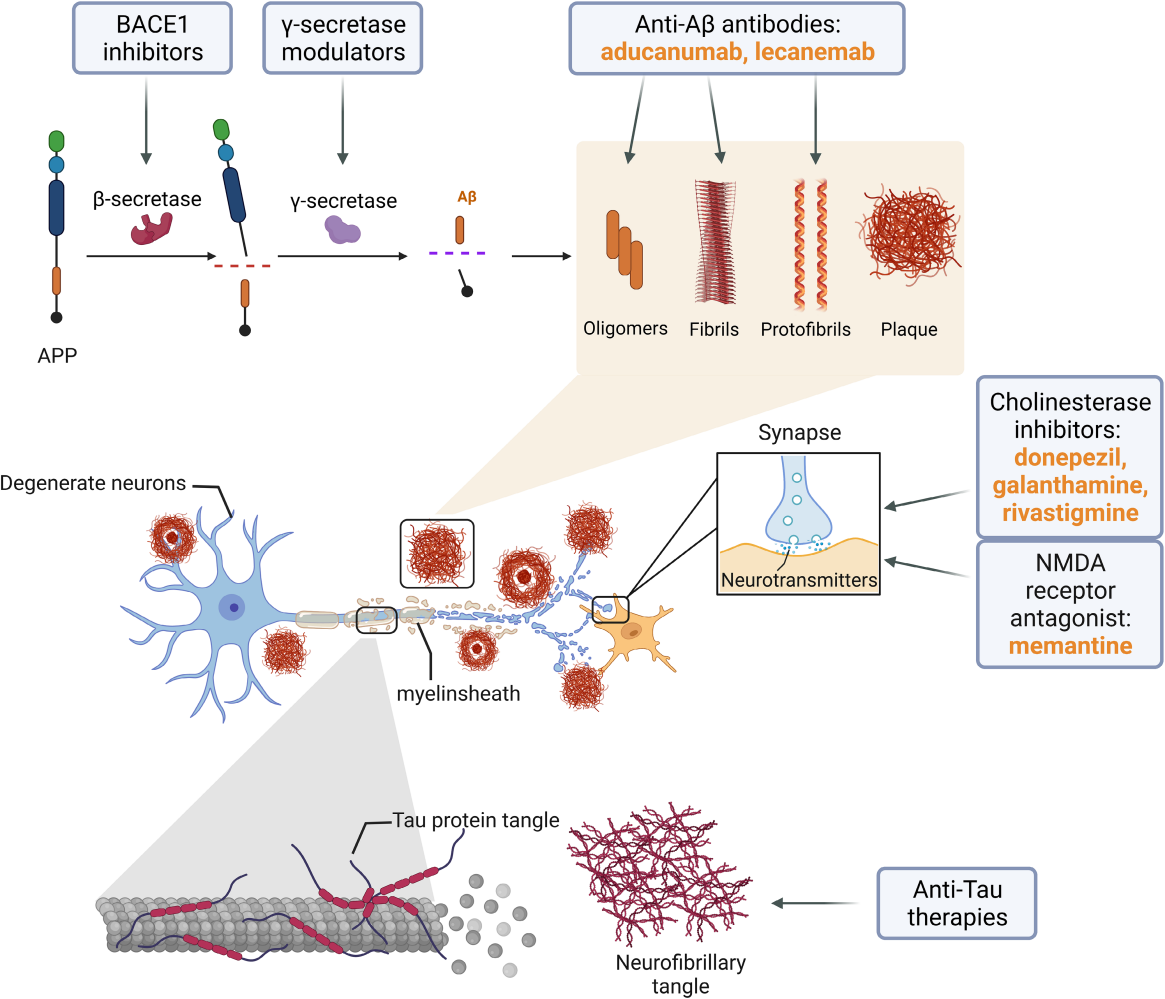
AD therapeutic agents and mechanisms. Three classes of drugs have been used to treat AD: cholinesterase inhibitors, NMDA receptor antagonist, and anti‐Aβ therapy. BACE1 inhibitors, γ‐secretase modulators, and anti‐Tau therapies are under investigation. APP, amyloid‐beta precursor protein; BACE1, beta‐secretase 1; Aβ, amyloid‐β; NMDA, N‐methyl‐D‐aspartic acid.

The dismal condition of AD therapy requires attention. Early detection and intervention could be possible solutions. Early diagnosis facilitates the identification of reversible causes, improves symptom management, and provides a future plan. In addition, based on the cost‐effectiveness analysis, the cost of early interventions is less than subsequent dementia treatment.^8^ Biomarkers have excellent prospects for early diagnosis. They are a class of molecules occurring in biological media, such as fluids or cells, and depict physiological states, pathological processes, or therapeutic agent responses. The significance of the biomarkers lies in: (1) Finding the latent disease to provide an opportunity for early intervention, particularly in patients having neurological disorders. (2) Providing some clues to molecular pathological mechanisms of AD, depending on the correlation between the appearance timing and changes in molecular biomarker levels and disease process. (3) Assisting in the differential diagnosis and determining the causes of the disease to select more targeted treatments, facilitating individualized treatment, and managing AD patients.^9^ (4) Developing new drugs based on the mechanisms and targets. AD drugs in the market and under investigation are presented in Figure 1 (developed using BioRender.com). Some biomarkers have already been identified and utilized in AD diagnosis, and more candidate biomarkers being investigated.

## 2 POTENTIAL BIOMARKERS OF AD

**FIGURE 2:**
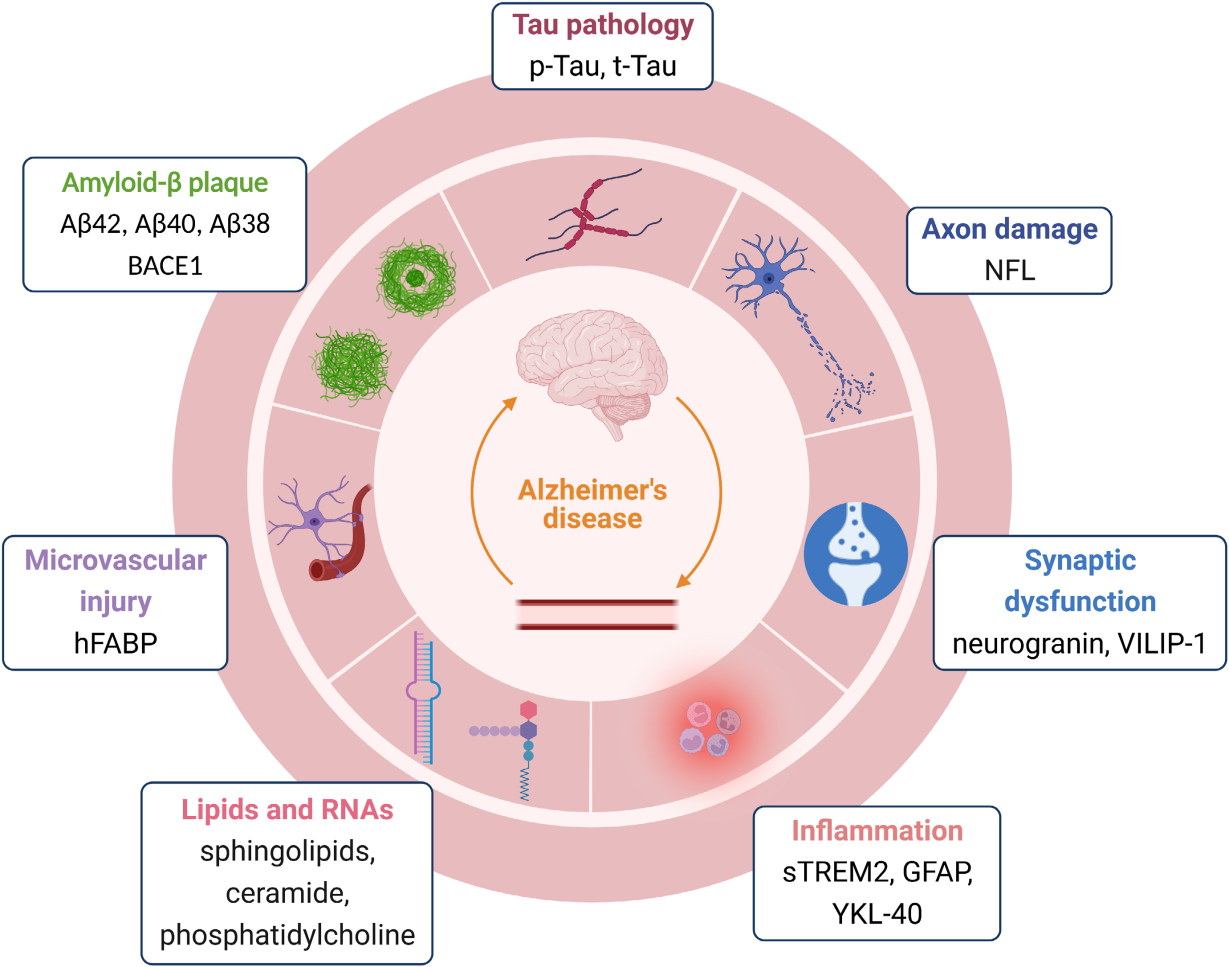
The biomarkers having a potential diagnostic and therapeutic value in body fluids identified so far have been primarily focused on the Aβ pathway, Tau pathology, inflammation, neuron axon‐associated molecules, synaptic dysfunction‐related elements, microvascular‐associated markers, lipids, and miRNAs. Aβ, amyloid‐β; BACE1, beta‐secretase 1; GFAP, glial fibrillary acidic protein; hFABP, heart‐type fatty acid‐binding protein; NFL, neurofilament light; sTREM2, soluble triggering receptor expressed on myeloid cells 2; VILIP‐1, visinin‐like protein‐1; YKL‐40/CHI3L1, chitinase 3‐like 1.

In 2007, cerebrospinal fluid (CSF) biomarkers were first included in the AD diagnostic criteria by the International Working Group, indicating their increasingly important role in diagnosing AD. The diagnosis of AD depends on the images, biomarkers, and scales.^10^ Positron emission tomography (PET) is the gold standard for AD diagnostics, and the high cost impedes its popularization. Scale tests are unable to distinguish and predict possible risk promptly. CSF biomarkers involve invasive extraction. In this case, other easily collectible fluids such as blood, urine, and saliva are the first choice to solve the difficulty. Several studies focus on diagnosing and predicting AD through fluid biomarkers, and many relevant test kits have been awaiting approval and marketing. The current study summarizes the most promising AD biomarkers for differentiating AD patients from healthy controls (Figure 2, developed using BioRender.com).

### 2.1 Biomarkers related to Aβ aggregation

Aβ is a polypeptide with 38–43 amino acids mainly produced by neurons. The most common human subtypes are Aβ_42_ with 42 amino acids and Aβ_40_ with 40 amino acids.^11^

Aβ is considered the initial trigger of AD, with research suggesting that Aβ is upstream of Tau for accelerating age‐related tauopathy and neurodegeneration.^11^, ^12^, ^13^ However, in 2020, a new study observed that subtle cognitive difficulties could be identified before or during the amyloid plaque deposition in the brain.^14^ This indicates that damage to neurons could happen before Aβ. Another likely reason is that brain scans could not detect the initial deposition of Aβ, demanding more evidence for validating the finding.

#### 2.1.1 Aβ peptide

The amount of Aβ or its fragments can facilitate an early AD diagnosis.^10^ Levels of CSF Aβ_42_ and Aβ_42_/Aβ_40_ both decrease in AD patients.^15^ A meta‐analysis revealed that the CSF concentration of Aβ_42_ was 0.55 times lower in AD cohorts than in healthy controls.^16^ Mild cognitive impairment (MCI) and dementia due to AD are associated with lower CSF levels of Aβ_42_. Compared with stable MCI, a 0.67 times reduction (95% CI 0.63–0.73, *p* < 0.001) was observed.^16^, ^17^ The CSF Aβ_42_/Aβ_40_ ratio decreased by 40%–60% in patients with cerebral Aβ pathology.^18^ Thus, the ratio of Aβ_42_/Aβ_40_ could predict the abnormal Aβ inside the brain and differentiate AD patients from normal individuals. Aβ_42_/Aβ_40_ holds promise for future applications but requires significant evidence to establish its sensitivity and specificity.

According to several studies, Aβ levels in blood and saliva showed significant changes with inconsistent results.^16^, ^19^ This could be due to the analytical sensitivity of the assay, as some early measurements were performed with enzyme‐linked immunosorbent assay (ELISA) methods. In contrast, recent studies utilized ultra‐sensitive immunoassays or targeted mass spectrometry (MS). Depending on the improvements in diagnostic techniques, more decreases of Aβ_42_ and Aβ_40_ levels in blood and saliva were observed in AD patients.^20^, ^21^, ^22^, ^23^

Aβ discovery offers new insights for developing drugs to combat AD. Anti‐Aβ antibody levels are lower than in the healthy population, indicating that the immune system could prevent the occurrence and development of AD.^24^, ^25^ Therefore, it becomes the theoretical basis of active immunotherapy. Phase 1 or 2 vaccine clinical trials are being conducted or completed. Unfortunately, no benefits were revealed, and many vaccines have significant adverse reactions.

Passive immunotherapy is promising, depending on current evidence. Aducanumab and lecanemab are humanized antibodies for reducing Aβ plaques and decreasing the risks of developing AD.^26^ They are already available in the U.S. amidst controversies. Dr. Knopman et al. described that no evidence was presented to correlate biomarker changes to cognitive benefits even though Aβ was decreased in aducanumab trials. *Post hoc* analysis indicates that the efficacy signal of aducanumab was unreliable.^27^ Definitive, optimally designed, and adequately powered trials should be undertaken to determine the efficacy of the drugs. On the contrary, the phase 3 double‐blinded trial on lecanemab satisfied all the primary and secondary endpoints, demonstrating significant improvements in brain amyloid and disease‐related scores. Further post‐marketing evaluation of its effectiveness is required.^28^ Both active and passive immunotherapeutic drugs targeting Aβ epitopes are represented in Table 1.

**TABLE 1:** Different target drugs for AD in clinical trials.

Therapeutic targets	Drug	Pharmaceutical companies	Main study outcomes and research status
Aβ	Aducanumab	Biogen	Aducanumab was approved for marketing in the United States in June 2021. There is great controversy about its efficacy.
	AFFITOPE AD01/AD02/AD03	AFFiRiS AG	No significant treatment effects were seen in clinical trials.^29^
	ALZ‐101	Alzinova AB	A Phase 1b study to evaluate tolerability, safety and immunological response to the vaccine is underway.
	AN1792	Janssen	Clinical trials stopped due to severe adverse events of meningoencephalitis.^30^
	Bacillus Calmette‐Guerin (BCG)	Steven E Arnold	The trial to evaluate the effects of BCG is recruiting.
	Bapineuzumab	Pfizer, Janssen	Two Phase 3 trials confirmed lack of efficacy of bapineuzumab in patients with mild to moderate AD.^31^ Bapineuzumab derivative was developed and showed good tolerability in Phase 1 trial.^32^
	CAD106	Novartis	CAD106 has favorable safety, tolerability, and acceptable antibody response in patients with AD.^33^ Efficacy has not been proven.
	Crenezumab	Hoffmann‐La Roche	Two Phase 3 trials of crenezumab versus placebo in early AD did not show efficacy.^34^ The study about efficacy in AD patients with PSEN1 mutation is underway.
	Donanemab	Eli Lilly and Company	Donanemab resulted in a better composite score for cognition and for the ability to perform activities of daily living than placebo in patients with early AD. A Phase 3 study of donanemab compared with aducanumab is ongoing.
	Gantenerumab	Hoffmann‐La Roche	Clinical trials stopped for futility.
	GSK933776	GlaxoSmithKline	Two Phase 1 trials were conducted but no further studies.
	Immune globulin		Participants with mild to moderate AD had no clinical benefit on cognition or function.^35^ Only a significant decrease in plasma Aβ_42_ was observed.
	Lecanemab	Eisai, Biogen	Lecanemab was approved for marketing in the United States in January 2023.
	Ponezumab	Pfizer	Clinical effect did not meet the expectation^36^ and the research on ponezumab was terminated.
	Solanezumab	Eli Lilly and Company	Solanezumab did not improve cognition of AD patients in a multicenter Phase 3 trial.^37^
	UB‐311	United Neuroscience	Patients with mild AD had a high responder rate.^38^ The primary outcome of efficacy is not available.
BACE1	Atabecestat	Janssen	Liver enzymes elevated and cognition declined in early AD patients treated with atabecestat.^39^
	Elenbecestat	Eisai, Biogen	People with elenbecestat had more loss of brain volume, loss of weight, liver enzymes elevation, neuropsychiatric and skin rashes adverse events than those on placebo.^40^ Elenbecestat resulted in a statistically significant reduction of brain amyloid.^40^
	Lanabecestat	AstraZeneca, Eli Lilly and Company	Clinical trials stopped for futility.
	LY2811376	Eli Lilly and Company	A Phase 1 trial was conducted but no further studies.
	LY2886721	Eli Lilly and Company	The study was terminated due to abnormal liver biochemical tests in some participants.
	Umibeestat	Novartis	Two clinical trials terminated due to cognitive worsening in treatment groups and safety issues.
	Verubecestat	Merck Sharp & Dohme	Verubecestat did not reduce cognitive decline in AD patients, whereas verbal fluency tasks showed improvement.^41^, ^42^
Tau	AADvac1	Axon Neuroscience SE	AADvac1 was well‐tolerated and had excellent immunogenicity, but the vaccine did not produce a statistically significant cognitive benefit.^43^
	ACI‐35 ACI‐35.030	AC Immune SA, Janssen	Results of Phase 1b trial of ACI‐35 were not published. ACI‐35.030 is the next iteration of ACI‐35. Phase 1/2 trial of ACI‐35.030 has no results so far.

#### Beta‐secretase 1 (BACE1)

BACE1 is another candidate biomarker for AD. It cleaves amyloid‐beta precursor protein in the first step of producing Aβ. CSF BACE1 activity and/or concentrations were significantly elevated in MCI. Thus, it could indicate an early AD stage, compared with healthy controls and AD.^44^, ^45^, ^46^ Cervellati et al. identified that BACE1 in serum changed across groups (*p* < 0.001) with a 25% increase in late‐onset AD against the controls. High BACE1 levels were independently associated with late‐onset AD diagnosis (odds ratios 2.79; 95% CI 1.37–5.67). This characteristic could identify AD during the pre‐dementia stage and predict its onset. The evidence supports the hypothesis that BACE1 is critical in AD pathophysiology, emerging as a new drug target in treating early AD to inhibit Aβ production.^47^ BACE1 inhibitors can slow down the progress of AD. Besides, some studies have also identified that inhibiting BACE1 stabilizes Tau levels in CSF,^48^, ^49^ although this is not its most significant role. Additionally, reduced brain volume and hepatic adverse reactions are common among patients using BACE1 inhibitors (Table 1).

### 2.2 Biomarkers related to Tau pathology

Tau, or microtubule‐associated protein tau (MAPT), is the primary component of the neuroaxonal protein bindings microtubules and stabilizing their assembly. Its function is dependent on the balance between phosphorylation and dephosphorylation. When Tau is hyperphosphorylated, the synthesis and assembly of microtubules are restricted. The aggregation of hyperphosphorylated Tau led to NFTs, inducing neuronal apoptosis.^50^, ^51^

Being the most significant characteristic of AD, Tau detection is essential for the early diagnosis of AD.^11^ Presently, total Tau (t‐Tau) and phosphorylation sites of Tau include threonine 181 (p‐Tau 181), serine 199 (p‐Tau 199), threonine 217 (p‐Tau 217), and threonine 231 (p‐Tau 231).^52^ The concentration of CSF t‐Tau is approximately three times higher in AD patients than in normal aging subjects.^53^ In blood, t‐Tau and p‐Tau also indicated a good association with AD. The study also observed that p‐Tau and t‐Tau are correlated with Tau‐PET signals within the temporal cortex (*r*_s_ > 0.56, *p* < 0.001) and predicted abnormal Tau‐PET status and Aβ.^54^ Moreover, AD diagnosis accuracy can be improved in combination with other biomarkers. The sensitivity and specificity were 85% and 95%,^55^ respectively, along with Aβ_42_. In saliva, t‐Tau and p‐Tau were detectable in participants having MCI, AD, and age‐matched controls. The p‐Tau levels were higher in AD without any statistical significance.^56^, ^57^, ^58^ Shi et al. observed a significant increase of 1.3‐folds in salivary p‐Tau 181/t‐Tau ratio in AD patients than in healthy controls. It was based on Luminex assays,^57^ a high throughput technology for simultaneous detection and quantification of multiple proteins, requiring more validations. Ultra‐sensitive assays are being refined for the reliable detection of salivary Tau. Thus, improving detective accuracy may identify more associations between salivary biomarkers.

The interest in therapeutic strategies targeting Tau is rising. Early anti‐Tau therapies were based on inhibiting kinases, Tau aggregation, or stabilizing microtubules. However, these approaches were terminated due to toxicity and/or lack of effectiveness.^59^ Besides, Tau immunotherapies are the topical strategy now. Currently, there are two vaccine regimens, AADvac1 and ACI‐35, under clinical active immunization trials (Table 1). AADvac1 binds a six‐amino‐acid sequence found in each of the microtubule‐binding repeats of Tau. Rats with tauopathy produced Tau antibodies after AADvac1 administration which reduced the level of pathological Tau, thereby improving the sensorimotor function scores in rats.^60^ In phase I trials, AADvac1 revealed good tolerance and safety without encephalitis or edema,^43^ with phase II trial under advanced development. ACI‐35 is used to target the phosphorylated residues Ser396 and Ser404 of Tau.^61^ A phase Ib/IIa clinical trial (NCT04445831) was initiated in 2019 to determine the safety and immunogenicity of ACI‐35.030 among early AD patients. Passive immunization is safer than active immunization, as patients do not produce these antibodies. Thus, passive immunization decreases the risk of adverse immunological effects. With many ongoing clinical trials, passive immunization also provides higher specificity for the epitope of interest.^59^

### 2.3 Biomarkers related to neuron axon

Neurofilaments (NFs) comprise three subunits: neurofilament light (NFL), medium, and heavy, and they are involved in axon formation. They maintain the integrity of neurons and conduct nerve impulses. When the axon is damaged, NFs are released into the CSF and eventually enter the bloodstream, specifically indicating axonal damage.

NFL has been reported as a potential biomarker in multiple sclerosis, AD, frontotemporal dementia, ALS, atypical Parkinson's disease (PD), and brain injury.^62^, ^63^ In AD patients, NFL levels are higher than in age‐matched controls in CSF and blood.^63^, ^64^ In addition to CSF and blood, the saliva NFL levels were determined by a single molecule array in a large study of 152 patients, with negative data. As with saliva Tau, NFL was detectable in MCI, AD, and healthy controls, which did not differ significantly.^65^

NFL is a sensitive but not a specific marker of axonal damage. NFL is unable to differentiate nervous system disorders characterized by axonal injury. However, it can evaluate the degrees of myelin sheath damage and the progression or severity of the disease, thereby assessing effective neuroprotective therapies.^66^, ^67^

### 2.4 Biomarkers related to synaptic dysfunction

Synaptic degeneration and dysfunction are early fundamental events of AD,^4^ directly causing the change and imbalance of neurotransmitters. The overstimulation of extra‐synaptic NMDA receptors and the associated synaptic redox stress leads to an influx of extracellular Ca^2+^. Therefore, a series of downstream pathways are initiated involving Tau phosphorylation.^51^

Neurogranin is a synaptic protein reflecting synaptic function and participates in AD pathophysiology.^68^ A meta‐analysis revealed that CSF neurogranin levels were significantly higher in MCI patients than in healthy controls (SMD 23.45, 95% CI 15.97–30.92, *Z* = 6.15, *p* < 0.001).^69^ CSF neurogranin is elevated in MCI patients who progressed to AD compared to patients with stable MCI. Considering the closely linked to cognitive impairment, Ann et al. identified some potential biomarkers and observed an association between neurogranin/BACE1 ratio and cognitive decline. The area under the receiver operating characteristics curve (AUC) was 0.715 (*p* = 0.008) in MCI patients and 0.806 (*p* < 0.001) in the AD group.^70^ Neurogranin/BACE1 ratio demonstrated a better prognostic value than the single analytes or other combinations.

Visinin‐like protein‐1 (VILIP‐1) is a neuronal Ca^2+^‐sensor protein acting as Ca^2+^‐dependent molecular switch. VILIP‐1 influences the intracellular neuronal signaling pathways involved in synaptic plasticities in the central nervous system, including cyclic nucleotide cascades and nicotinergic signaling.^71^ VILIP‐1 is higher in the CSF of AD patients than in healthy controls. The ratio between AD and controls of VILIP‐1 was analyzed to depict an average ratio of 1.46 (95% CI 1.31–1.62, *p* < 0.001).^16^ Some studies indicated that VILIP‐1 was closely related to Tau and Aβ_42._ ^72^, ^73^, ^74^ Researchers attempted to detect VILIP‐1, Tau, and Aβ simultaneously and found that CSF levels of VILIP‐1/Aβ_42_ could predict future cognitive impairment and AD diagnosis with satisfactory accuracy.^75^

Other synaptic‐related proteins reflect the physiological synaptic functions. These include human synaptosomal‐associated protein‐25 (SNAP‐25), growth‐associated protein‐43 (GAP‐43), and synaptotagmin, as potential AD biomarkers. A correlation between these modules and AD was found without much specificity.^76^ More studies are required to validate the relationship between synaptic‐related proteins and AD.

### 2.5 Biomarkers related to inflammation

Aβ activates various cell receptors and signaling pathways causing inflammation. Microglia and astrocytes are active in neuroinflammation. Thus, several biomolecules generated by these cells could become promising AD biomarkers.

#### 2.5.1 Triggering receptor expressed on myeloid cells 2 (TREM2)

TREM2 is a transmembrane glycoprotein, highly expressed in microglia and strongly associated with Tau and inflammation.^77^, ^78^ TREM2 can be activated by anti‐inflammatory factors and inhibited by some pro‐inflammatory cytokines such as interleukin‐1β (IL‐1β), IL‐6, and tumor necrosis factor‐α (TNF‐α). Its precise role in inflammation and Tau pathology is unclear. However, overexpression of TREM2 can rescue spatial cognitive impairment and improve neuropathology, such as neuronal and synaptic loss and Tau hyperphosphorylation.^77^, ^79^ Soluble TREM2 (sTREM2) is a TREM2 fragment detected in CSF, is age‐related, and positively correlates with neurodegenerative biomarkers including Aβ_42_, p‐Tau, and t‐Tau. Ewers et al. examined CSF sTREM2 in 385 elderly subjects involving cognitively normal controls, MCI individuals, and AD dementia patient. A strong correlation was observed between high CSF sTREM2 levels and reduced cognitive decline at different AD stages.^80^ Thus, sTREM2 appears to be applied as a prognosis biomarker and provides a perspective for therapeutic drug targets on AD immune response. Several studies also observed a moderate elevation in CSF sTREM2 levels in AD patients.^81^ The peak occurred in the early symptomatic phase, with a slight decline in the clinical dementia stage of AD.^82^ A meta‐analysis revealed that CSF sTREM2 levels in AD patients were higher than in healthy subjects, suggesting that it could be utilized as a biomarker to diagnose or determine the clinical stages of AD.^83^ High levels of sTREM2 can also be observed in other neurodegenerative diseases despite the apparent increase of sTREM2 in AD. Clarifying its role in AD or complementarity with other specific biomarkers could facilitate its application in AD.

#### 2.5.2 Glial Fibrillary Acidic Protein (GFAP)

GFAP is a specific brain protein expressed by astrocytes and involved in their structure and movement. Some research demonstrated that astrocytes could respond to neuroinflammation and GFAP has a concomitant change.^84^ An elevated GFAP in CSF has been observed in AD, PD, Lewy body dementia (LBD), frontotemporal dementia, and prion disease.^85^, ^86^ Moreover, there is an increase in plasma GFAP in AD, PD dementia, and LBD. However, more studies could not show the prominent differences between neurological disorders. The average ratio of AD to controls in CSF was 1.12 (95% CI 0.58–2.15, *p* = 0.736).^16^ Thus, GFAP is not a promising CSF diagnostic biomarker for AD. The inconsistent GFAP changes may be associated with the different affected brain regions and astrocytes distribution. The sensitivity and specificity of serum GFAP differentiating AD patients from healthy age‐matched controls are high, reaching 77% and 93%, respectively. These two GFAP indexes are even higher than Aβ_42_ and slightly lower than Tau.^85^, ^87^ Additionally, salivary GFAP levels were determined using ELISA by Katsipis et al. GFAP concentrations were lower in MCI (6.82 ± 2.10 ng/mL) and AD (3.56 ± 2.24 ng/mL) than in cognitively healthy subjects (13.35 ± 3.03 ng/mL). Statistically, significant differences were observed in the levels compared to each other (*p* < 0.001).^88^ This finding indicated that salivary GFAP could be an excellent biomarker for discriminating controls from MCI or AD patients.

The poor specificity in CSF limits GFAP usage as a biomarker. However, GFAP is a promising candidate peripheral biomarker providing disease‐specific information, considering blood and saliva.^89^ Several large‐scale studies are required to validate this conclusion.

#### 2.5.3 Chitinase 3‐like 1 (CHI3L1/YKL‐40)

YKL‐40 is a 4 kDa glycoprotein, also called CHI3L1. This glycoprotein is secreted by microglia; its CSF content is positively associated with t‐Tau and p‐Tau,^90^ while it is negatively correlated with AD progression.^91^ YKL‐40 increases in CSF and decreases in the serum of AD patients.^90^, ^92^ YKL‐40, along with sTREM2 in young subjects, revealed better results in predicting AD.^93^ Therefore, YKL‐40 may be a potential biomarker to predict AD occurrence.

### 2.6 Biomarkers related to microvascular injury

Cerebrovascular disease was reported more commonly in AD than in other neurodegenerative disorders.^94^ Vascular dysregulation plays an important role in AD, even though the molecular pathogenesis of vascular alterations remains undetermined. Some proofs suggested that microvascular injury was associated with disease onset and progression, reducing oxygen and nutrient supply to the brain.^95^ This led to subsequent neurotoxic effects, such as inflammation, oxidative stress, and nitric oxide disorder.^96^

Vascular dysregulation is related to abnormally high heart‐type fatty acid‐binding protein (hFABP) levels.^97^ It is a potential cardiac damage biomarker for predicting acute myocardial infarction, congestive heart failure, or pulmonary embolism.^98^ However, the hFABP levels also increased in CSF and plasma in AD. Therefore, it can be a potential biomarker for predicting and differentiating against AD.^99^ Chiasserini et al. demonstrated that the combination of hFABP with p‐Tau led to high accuracy in the differential diagnosis of AD from LBD with 76% specificity and 95% sensitivity (AUC = 0.92).^100^ Lehallier et al. observed that a combination of hFABP, fibroblast growth factor 4, calcitonin, and tumor necrosis factor‐related apoptosis‐inducing ligand receptor 3 in CSF, apolipoprotein A‐II and cortisol levels in plasma enhanced the reliability of disease status prediction with 80% classification accuracy, 88% sensitivity, and 70% specificity.^101^ More combinations and values remain to be explored in the future.

### 2.7 Biomarkers related to heredity

Amyloid‐beta precursor protein, presenilin 1, and presenilin 2 are genes correlated with early‐onset AD, especially familial ones. In contrast, the late‐onset of AD is more closely associated with other genes, including apolipoprotein E (ApoE), bridging integrator1 (BIN1), and cluster protein (CLU). The ApoE‐ε4 allele is the most promising candidate gene biomarker and possesses serious genetic risk factors for AD.^15^

Astrocytes in the central nervous system produce ApoE. The more copy numbers of the ApoE‐ε4 gene, the higher the susceptibility to AD.^102^ The increase in ApoE impairs cholesterol transmission between astrocytes and neurons. This led to cholesterol accumulation in the neurons, affecting the clearance rate of Aβ and increasing its production.^15^ ApoE plays various biochemical roles in the brain, including regulating immune cells and Tau tangles. However, the specific mechanism of ApoE has not been fully elucidated in this context.^102^

### 2.8 Others

#### 2.8.1 Biomarkers associated with lipid metabolism

Lipids, including phospholipids, cholesterol, sphingolipids, and related molecules, are the primary component of cell membranes, and the brain is enriched in lipids.^103^ Sixty percent of the non‐aqueous portion of the brain comprises cerebral lipids. Thus, changes in lipid metabolism could influence brain function.^104^ Alterations of fatty acids and cerebral lipid peroxidation were observed in the early AD stage. A higher level of lipid particles in glial cells was seen in the brain of AD patients, indicating abnormal lipid metabolism.^103^ Several genes, like ApoE, are also involved in lipid homeostasis.^102^ Lipid evaluation analyzes body fluids using highly sensitive MS platforms and high‐throughput technologies. Therefore, it has become one of the interests in the search for AD biomarkers.

A few studies determined the lipid changes in CSF and blood. Several lipid families (fatty acid, sphingolipids, glycerophospholipids, and lipid peroxidation compounds) demonstrated impairments at early AD stages. In contrast, ceramides were elevated compared with healthy age‐matched controls.^105^, ^106^ A single lipid could not predict or diagnose diseases because the changes in lipids metabolism were common in multiple neurological conditions. García‐Blanco et al. attempted to identify a new set of lipid peroxidation compounds in urine samples. 17(RS)‐10‐epi‐SC‐Δ15‐11‐dihomo‐IsoF, prostaglandin E2 (PGE2), neuroprostanes, isoprostanes, and isofurans revealed the difference between patients and healthy controls by using ultra‐performance liquid chromatography‐tandem MS.^106^ This indicated that lipids panels could be potential early AD biomarkers, although these indicators, such as PGE2, vary in the urine of patients with other diseases. Additional research is required to validate the changes and identify the role of lipids families in AD.

#### 2.8.2 Biomarkers related to RNA

In addition to exploring AD‐related proteins and lipids, RNA has also gained attention in recent years. RNAs are essential molecules that regulate protein transcription and translation processes before protein synthesis.^107^

MicroRNAs (miRNAs) are small non‐coding RNA with 22 ~ 24 nucleotides. MiRNAs can regulate the expression of genes by binding to the 3′‐untranslated region (3′UTR) of messenger RNA (mRNA). Changes in miRNAs reflect abnormal alterations in downstream proteins in different pathways, implicated in various AD stages.^107^ Many studies showed that some kinds of miRNAs are significantly deregulated in disease groups than in control groups.^108^ A systematic review summarized a panel of 10 miRNAs, including miR‐107, miR‐125b, miR‐146a, miR‐26b, miR‐200c, miR‐210, miR‐30e, miR‐34a, miR‐485, and miR‐34c, which were associated with early AD.^109^ However, further research is needed to determine the application of miRNA biomarkers due to conflicting miRNA variations or inconsistency in reported results.^110^, ^111^

The main biomarkers of AD are summarized in Table 2.

**TABLE 2:** Candidate biomarkers in AD patients compared to healthy controls.

Pathology	AD biomarkers	CSF	Serum/plasma	Saliva	References
Aβ deposition	Aβ_42_	↓	↓ ^a^	↑^a^	19, 112
	Aβ_40_	↓^a^	↓^a^	—	19, 113, 114
	Aβ_42_/Aβ_40_	↓	↓	NR	112, 115, 116
	Aβ_38_	—^a^	^a^	NR	116, 117, 118, 119
	BACE1	↑^a^	↑	NR	44, 120
Tau pathology	t‐Tau	↑	↑	—	16, 19, 112, 113
	p‐Tau	↑	↑	—^a^	112, 113, 121, 122
Axon damage	NFL	↑	↑	—	63, 64, 65, 99, 123, 124
Synaptic dysfunction	Neurogranin	↑^a^	↑^a^	NR	69, 75, 125
	VILIP‐1	↑^a^	↑	NR	72, 74, 75, 124
Inflammation	sTREM2	↑^a^	—	NR	^83^
	GFAP	↑^a^	↑	↓	85, 86, 88, 126
	YKL‐40	↑^a^	↑	NR	91, 124, 125, 127
Microvascular injury	hFABP	↑	—	NR	51, 99, 128, 129
Lipid metabolism	Sphingolipids	↑	—^a^	NR	130, 131, 132
	Ceramide	↑	↑	NR	133, 134, 135
	Phosphatidylcholine	↓	↓	NR	136, 137, 138, 139
RNA	miRNA	Data are limited. Further studies are needed			

Abbreviations: Aβ, amyloid‐β; BACE1, Beta‐secretase 1; GFAP, glial fibrillary acidic protein; hFABP, heart‐type fatty acid‐binding protein; miRNA, microRNA; NFL, neurofilament light; NR: not reported; sTREM2, soluble triggering receptor expressed on myeloid cells 2; VILIP‐1, visinin‐like protein‐1; YKL‐40, chitinase 3‐like 1.

^*a*^ Inconsistent results exist or controversies remain.

## 3 BIOINFORMATICS: TOOLS FOR FINDING BIOMARKERS

The discovery of biomarkers depends on detection and bioinformatics technology. Biomarkers of neurological diseases possess low concentrations in CSF and peripheral fluids, demanding higher sensitivity, accuracy, and instrument resolution.^140^ Hence, utilizing suitable detection methods is necessary to discover effective biomarkers. Ultra‐sensitive immunoassays and targeted MS, such as ELISA, single‐molecule array, immunoprecipitation coupled with MS, and stable isotope labeling kinetics after immunoprecipitation associated with MS, have been widely utilized for identifying biomarkers.^141^ ELISA kits are commercialized, facilitating significant biomarker tests in clinical practice. Application and diagnostic kits of AD biomarkers are summarized in Table 3.

**TABLE 3:** Application and diagnostic kits of AD biomarkers.

AD biomarkers	Potential application	diagnostic kit
Aβ_42_	·Diagnostic capacity is confirmed ·Anti‐Aβ antibody and vaccines are under investigation ·Aducanumab and lecanemab are approved for therapy by FDA	Elecsys B‐Amyloid (1–42) CSF II is approved for diagnostic test by FDA
Aβ_40_		Commercial kits for scientific research^142^
Aβ_42_/Aβ_40_		Lumipulse G β‐Amyloid Ratio (1–42/1–40) is approved for diagnostic test by FDA
Aβ_38_	·To evaluate cognitive decline ·γ‐secretase modulators is under investigation	Commercial kits for scientific research^143^
BACE1	·To predict and identify AD in the pre‐dementia stage ·BACE1 inhibitors are under investigation	Commercial kits for scientific research^144^
t‐Tau	·Diagnostic capacity is confirmed ·Anti‐Tau therapies are under investigation	Commercial kits for scientific research^145^
p‐Tau		Elecsys Phospho‐Tau (181P) CSF is approved for diagnostic test by FDA
NFL	·To evaluate axonal damage and disease progression ·To diagnose neurodegenerative disease of Alzheimer type associated with AD phenotype	Commercial kits for scientific research^146^
Neurogranin	·To prognose the disease	Commercial kits for scientific research^147^
VILIP‐1	·To predict cognitive impairment	Commercial kits for scientific research^148^
sTREM2	·To identify the stages of AD ·To Adjunct to other biomarkers	Commercial kits for scientific research^93^
GFAP	·To differential diagnose	Commercial kits for scientific research^126^
YKL‐40	·To predict AD	Commercial kits for scientific research^149^
hFABP	·To discriminate and predict AD ·Adjunct with other biomarkers	Commercial kits for scientific research^150^
Sphingolipids	·To Diagnose AD at early stage	Commercial kits for scientific research^104^
Ceramide		Commercial kits for scientific research^104^
Phosphatidylcholine		Commercial kits for scientific research^104^
miRNA	·Remain to be explored	Commercial kits for scientific research^151^

Bioinformatics combines information software and assay technology for researchers to store, retrieve, visualize, predict, and analyze biomolecular data, significantly improving research efficiency. Omics, including proteomics, lipidomics, and RNomics were established based on bioinformatics. They enable the identification of the underlying molecules of the disease with increased comprehension, leading the way toward diagnosis and precision medicine.^140^, ^141^ Future studies must exploit biomarkers discovered by bioinformatics to achieve clinical translation.

## 4 CONCLUSIONS

Biomarkers exploration is essential in detecting diseases because it can help predict, diagnose, identify, treat diseases, and understand disease processes. In 2021, Aβ_42_, t‐Tau, p‐Tau, and NFL were incorporated into the diagnostic criteria of AD, marking a significant breakthrough in studying biomarkers for neurological disorders. Other biomarkers, including inflammation‐related and synaptic function‐related biomarkers, are promising for future clinical implications due to their confirmed pathologies in AD. However, since the pathological phenomenon behind microvascular damage is not fully established in AD and could only be a secondary lesion in the late stage, its significance as a biomarker for early diagnosis may be minimal.^96^ Similar changes occur in many neurological diseases, and the specificity is not high in AD, even though lipid metabolites may be a new direction.^135^ Thus, the possibility of utilizing them as early diagnostic markers for AD is doubtful. MiRNA has been a hot area recently since its length is too short and its nucleotide sequences are not specific.^107^ However, the accuracy of precise identification of one particular RNA needs to be enhanced, and the combined detection results of multiple molecules would probably be more reliable. Nevertheless, the application prospect of such biomarkers is also worrying. The common issues with many of them require an urgent solution.

First, some studies on the same potential biomarkers indicated divergent or opposite results (Table 2). Therefore, more in‐depth studies are needed to determine the reliability of novel biomarkers.

Second, the sensitivity and specificity of many candidate biomarkers have not been satisfactory enough. To address this issue, scientists have actively experimented strategies that use multiple potential biomarkers, evaluating the relationship between various biomarkers and diseases and gauging whether diagnostic accuracy can be improved using multiple biomarkers. Some results have been achieved. For instance, the ratio of Aβ_42_/Aβ_40_ has superior performance than Aβ_42_ alone.^115^ The increase in the neurogranin/BACE1 ratio predicts cognitive decline.^70^, ^152^ The concentrations of YKL‐40 and sTREM inflammatory biomarkers are closely associated with the changes in brain imaging. Thus, combining inflammatory biomarkers with imaging can improve the diagnostic yield among subjects.^93^ A good combination of biomarkers can enhance the specificity and sensitivity of diseases, thereby compensating for the shortcomings of a single biomarker in practical application.

The third problem is sampling. CSF and blood mainly represent current samples for biomarkers. CSF sampling is more complex, expensive, and invasive than blood, making it unsuitable for repeat operations in a short time. In contrast, blood sampling avoids the above problems with higher clinical feasibility. Inevitably, blood samples can be contaminated with trace amounts of protein or lipids. Hence, these impurities should be accommodated when setting the range for abnormal values. Nevertheless, exploring blood biomarkers in neurological diseases should be the focus of future research.^153^

In addition, the discovery of biomarkers also provides direction and ideas for developing new drugs. Targeted agents based on Aβ, BACE1, and Tau are in clinical trials, and aducanumab and lecanemab are already available in the market. These compounds have significant potential to become new drugs for AD treatment.

Although there is little convincing evidence that current biomarkers are sufficiently specific and sensitive to diagnose AD, the potential socioeconomic and medical benefits of developing drugs to treat AD still make the search for biomarkers a fruitful area of study.

## AUTHOR CONTRIBUTIONS

Z.G. Zhao and G.H. Du conceived and designed the review. Y.N. Xu, H.L. Jiang, Z.S. Sun, and T. Feng searched and collected literature information. H.L. Jiang, B. Zhu, and M.N. Cao draw the figure and organized the tables. Y.N. Xu wrote the manuscript draft. Z.G. Zhao and H.L. Jiang are the principal investigators of the funds. Z.G. Zhao, G.H. Du, and H.L. Jiang revised and edited the final manuscript. All authors read and approved the final manuscript.

## CONFLICT OF INTEREST STATEMENT

The authors have no conflicts of interest to declare.

## Data Availability

Data sharing does not apply to this article, and this research does not involve the analysis and innovation of new data.
